# Impact of non-cystic fibrosis bronchiectasis on critically ill patients in Korea: a retrospective observational study

**DOI:** 10.1038/s41598-021-95366-z

**Published:** 2021-08-03

**Authors:** Youngmok Park, Seung Hyun Yong, Ah Young Leem, Song Yee Kim, Sang Hoon Lee, Kyungsoo Chung, Eun Young Kim, Ji Ye Jung, Young Ae Kang, Moo Suk Park, Young Sam Kim, Su Hwan Lee

**Affiliations:** Division of Pulmonary and Critical Care Medicine, Department of Internal Medicine, Severance Hospital, Yonsei University College of Medicine, 50-1 Yonsei-ro, Seodaemun-gu, Seoul, 03722 Republic of Korea

**Keywords:** Respiratory tract diseases, Prognosis

## Abstract

This study investigated the impact of bronchiectasis on patients admitted to the intensive care unit (ICU) at a hospital in Korea. Patients with bronchiectasis were diagnosed using results of chest computed tomography performed before ICU admission. The severity of bronchiectasis was based on the number of affected lobes, and patients with ≥ 3 bronchiectatic lobes were classified into the severe bronchiectasis group. Overall, 823 patients were enrolled. The mean age was 66.0 ± 13.9 years, and 63.4% were men. Bronchiectasis and severe bronchiectasis were present in 148 (18.0%) and 108 (13.1%) patients, respectively. The increase in the number of bronchiectatic lobes was related to the rise in ICU mortality (*P* for trend = 0.012) and in-hospital mortality (*P* for trend = 0.004). Patients with severe bronchiectasis had higher odds for 28-day mortality [odds ratio (OR) 1.122, 95% confidence interval (CI) 1.024–1.230], ICU mortality (OR 1.119, 95% CI 1.023–1.223), and in-hospital mortality (OR 1.208, 95% CI 1.092–1.337). The severe bronchiectasis group showed lower overall survival (log-rank *P* < 0.001), and the adjusted hazard ratio was 1.535 (95% CI 1.178–2.001). Severe bronchiectasis had a negative impact on all-cause mortality during ICU and hospital stays, resulting in a lower survival rate.

## Introduction

Bronchiectasis is characterized by persistent dilatation of the bronchi^1^. It impairs the clearance of bacteria and mucus from the airway, leading to progressive airway damage, decreased lung function, respiratory failure, and death^2^. The true prevalence of bronchiectasis is unknown. It does seem to vary geographically, but there are many confounders to this: notably, access and ready use of computed tomography (CT), which is needed for diagnosis^3–5^. Patients with bronchiectasis are known to have multimorbidity^6^, experience frequent exacerbation^7^, and present twofold increased mortality compared to the general population^4^.

Although several studies have reported the impact of non-cystic fibrosis bronchiectasis (hereafter bronchiectasis) on the general population, only a few studies have explored its impact on critically ill patients^8–10^. Dupont et al.^8^ reviewed 48 bronchiectasis patients admitted in the medical intensive care unit (ICU) and found that age > 65 years and prior use of long-term oxygen therapy negatively affected the survival. Alzeer et al.^9^ presented similar results and found that age over 65 years, low physical activity, and the use of inotropic support were risk factors of mortality in bronchiectasis patients. A study from the UK reported worse mortality rates in the ICU and hospital among bronchiectasis patients than among patients with chronic obstructive pulmonary disease (COPD) in the elderly population^10^.

However, the impact of bronchiectasis on critically ill patients is not fully understood, and, to the best of our knowledge, data from Korea have not been previously reported. Therefore, our study aimed to investigate the impact of bronchiectasis on patients admitted to the medical ICU at a tertiary referral hospital in Korea. The primary outcome was all-cause mortality assessed at 28 days after ICU admission, ICU mortality, and in-hospital mortality. The secondary outcomes were the durations of ICU and hospital stay and overall survival.

## Material and methods

### Study design and population

This retrospective cohort study enrolled patients admitted to the 24-bed medical ICU of Severance Hospital, a tertiary referral hospital in Seoul, Korea, from July 1, 2016, to April 30, 2019. The study was conducted according to the guidelines of the Declaration of Helsinki, and the study protocol was approved by the institutional review board of Severance Hospital (IRB No. 4-2020-1098). The committee waived the need for informed consent from the patients in consideration of the retrospective nature of the present study.

According to our institutional policy, patients with acute coronary disease are admitted to a coronary care unit. Patients requiring immediate postoperative management are also admitted to the surgical ICU. Accordingly, patients requiring mechanical ventilation or continuous renal replacement therapy were admitted to the medical ICU in the current study.

Patients who were aged > 18 years and underwent chest CT before ICU admission were considered eligible for this study. Patients without a chest CT scan taken prior to ICU admission or without an official CT report by thoracic radiologists were excluded.

### Data collection and study outcome

The demographic data, laboratory findings, comorbidities, and hospital course were obtained from electronic medical records; these data included age, sex, body mass index (BMI), a history of COPD or asthma (from clinical diagnosis made by respiratory physicians, spirometry results, and prescription of inhalers), and the Charlson comorbidity index (CCI)^11^, Acute Physiology and Chronic Health Evaluation II (APACHE II)^12^, and Sequential Organ Failure Assessment (SOFA) scores^13^. Mechanical ventilatory support and septic shock, based on the Third International Consensus definitions, were also investigated^14^. Survival data were acquired until June 30, 2020.

### Definition of bronchiectasis and radiologic severity

Bronchiectasis, including traction bronchiectasis and bronchiolectasis, were detected using a chest CT scan before ICU admission. The radiological extent of bronchiectasis, evaluated by two independent pulmonary physicians, was used to represent disease severity according to the number of affected lobes; the presence of three or more bronchiectatic lobes out of a total of five lobes was considered to represent severe bronchiectasis^15–17^. Patients with non-cystic fibrosis bronchiectasis were enrolled in this study because very few cases of infants with cystic fibrosis have been reported in Korea.

### Statistical analyses

Categorical variables were analyzed using Pearson’s chi-squared test or Fisher's exact test, and continuous variables were analyzed with Student’s *t*-test or the Mann–Whitney U test. The Cochran-Armitage trend test was used for comparing changing trends. The odds ratio (OR) and 95% confidence interval (CI) for all-cause mortality were measured with logistic linear regression analysis. Survival analysis was also performed: overall survival with the Kaplan–Meier method and the statistical difference with the log-rank test. Hazard ratio (HR) and 95% CIs were evaluated with the Cox proportional-hazards model. All statistical analyses were conducted using the R program v4.0.2 (The R Foundation for Statistical Computing, Vienna, Austria), and a two-tailed *P*-value of < 0.05 was considered statistically significant.

## Results

### Baseline characteristics

Overall, 1207 patients were admitted to the ICU during the study period, and the eligibility screening identified 823 (68.2%) patients. The median interval between ICU admission and chest CT was 3 days (interquartile range: 0–24 days). The population's baseline characteristics are presented in Table 1. There were 148 (18.0%) patients with bronchiectasis and 108 (13.1%) patients with severe bronchiectasis. There was no significant difference in the APACHE II score of the enrolled patients with or without severe bronchiectasis. However, the initial SOFA score was lower in the group with severe bronchiectasis than in the group without (7.7 vs. 8.8, *P* = 0.009). In the severe bronchiectasis group, the lengths of ICU stay (13 days vs. 8 days, *P* < 0.001) and hospital stay (47 days vs. 35 days, *P* = 0.037) were longer and intubation rates (85.2% vs. 68.3%, *P* < 0.001) were higher.

**Table 1:** Baseline characteristics and outcomes of the study population.

	Total (n = 823)	Without severe Bronchiectasis (n = 715)	Severe Bronchiectasis (n = 108)	*P-*value
Age, years	66.0 ± 13.9	66.1 ± 14.1	65.1 ± 12.6	0.493
Sex, male	522 (63.4)	458 (64.1)	64 (59.3)	0.391
BMI, kg/m^2^	22.1 ± 4.4	22.4 ± 4.4	20.6 ± 3.8	< 0.001
APACHE II score	25.7 ± 9.0	25.6 ± 9.0	26.4 ± 9.1	0.439
SOFA score	8.6 ± 3.8	8.8 ± 3.8	7.7 ± 3.3	0.009
CCI score	3.7 ± 2.5	3.7 ± 2.5	3.5 ± 2.7	0.598
Asthma	52 (6.3)	40 (5.6)	12 (11.1)	0.047
COPD	99 (12.0)	71 (9.9)	28 (25.9)	< 0.001
Bronchiectasis	148 (18.0)			
Severe bronchiectasis	108 (13.1)			
Intubation	580 (70.5)	488 (68.3)	92 (85.2)	< 0.001
Septic shock	588 (71.4)	514 (71.9)	74 (68.5)	0.543
CRRT	294 (35.7)	266 (37.2)	28 (35.9)	0.030
**Outcomes**				
28-day mortality	236 (28.7)	196 (27.4)	40 (37.0)	0.052
ICU mortality	215 (26.1)	176 (24.6)	39 (36.1)	0.016
In-hospital mortality	358 (43.5)	294 (41.1)	64 (59.3)	0.001
ICU stay, days	8 [4–17]	8 [4–17]	13 [6–22]	< 0.001
Hospital stay, days	36 [18–71]	35 [17–69]	47 [23–90]	0.037
Follow-up, months	2.6 [0.6–14.9]	2.8 [0.6–15.7]	2.0 [0.6–10.0]	0.109

*Note*: Data are presented as numbers (%), mean ± standard deviation, or median [interquartile range].

*APACHE II* Acute Physiology and Chronic Health Evaluation II, *BMI* body mass index, *CCI* Charlson comorbidity index, *COPD* chronic obstructive pulmonary disease, *CRRT* continuous renal replacement therapy, *ICU* intensive care unit, *SOFA* sequential organ failure assessment.

We performed a subgroup analysis of patients with respiratory failure requiring intubation and mechanical ventilation (Table 2). The prevalence of bronchiectasis and severe bronchiectasis was 21.6% and 15.9%, respectively. The initial SOFA score was also lower in the severe bronchiectasis group (7.9 vs. 9.0, *P* = 0.006), but the length of ICU and hospital stays was similar between the two groups.

**Table 2:** Baseline characteristics and outcomes of intubated patients.

	Total (n = 580)	Without severe Bronchiectasis (n = 488)	Severe Bronchiectasis (n = 92)	*P*-value
Age, years	66.1 ± 13.9	66.4 ± 14.1	64.7 ± 13.0	0.267
Sex, male	383 (66.0)	328 (67.2)	55 (59.8)	0.208
BMI, kg/m^2^	22.0 ± 4.4	22.2 ± 4.5	20.7 ± 3.7	0.001
APACHE II score	27.6 ± 8.6	27.8 ± 8.6	26.8 ± 9.1	0.321
SOFA score	8.8 ± 3.9	9.0 ± 4.0	7.9 ± 3.2	0.006
CCI score	3.6 ± 2.5	3.6 ± 2.5	3.5 ± 2.7	0.666
Asthma	45 (7.1)	35 (7.2)	10 (10.9)	0.316
COPD	83 (14.3)	56 (11.5)	27 (29.3)	< 0.001
Bronchiectasis	125 (21.6)			
Severe bronchiectasis	92 (15.9)			
Septic shock	435 (75.0)	367 (75.2)	68 (73.9)	0.896
CRRT	179 (30.9)	156 (32.0)	23 (25.0)	0.229
**Outcomes**				
28-day mortality	192 (33.1)	157 (32.2)	35 (38.0)	0.329
ICU mortality	190 (32.8)	153 (31.4)	37 (40.2)	0.123
In-hospital mortality	292 (50.3)	235 (48.2)	57 (62.0)	0.021
ICU stay, day	12 [6–21]	11 [6–21]	14 [8–24]	0.056
Hospital stay, day	40 [20–74]	40 [20–72]	50 [25–108]	0.055

*Note*: Data are presented as numbers (%), mean ± standard deviation, or median [interquartile range].

*APACHE II* Acute Physiology and Chronic Health Evaluation II, *BMI* body mass index, *CCI* Charlson comorbidity index, *COPD* chronic obstructive pulmonary disease, *CRRT* continuous renal replacement therapy, *ICU* intensive care unit, *SOFA* sequential organ failure assessment.

### Bronchiectasis and mortality

The crude 28-day mortality, ICU mortality, and in-hospital mortality rates were 28.7%, 26.1%, and 43.5%, respectively (Table 1). Patients with severe bronchiectasis had higher rates of ICU mortality (36.1% vs. 24.6%, *P* = 0.016) and in-hospital mortality (59.3% vs. 41.1%, *P* = 0.001) than did those without severe bronchiectasis. As more lobes were involved in bronchiectasis, higher rates of ICU mortality (*P* for trend = 0.012) and in-hospital mortality (*P* for trend = 0.004) were observed (Table 3).

**Table 3:** Number of bronchiectatic lobes and mortality.

	No. of Bronchiectatic Lobes						
	0	1	2	3	4	5	*P* for trend
**All patients (n = 823)**	(n = 675)	(n = 16)	(n = 24)	(n = 22)	(n = 24)	(n = 62)	
28-day mortality	187 (77.7)	5 (31.2)	4 (16.7)	7 (31.8)	8 (33.3)	25 (40.3)	0.058
ICU mortality	169 (25.0)	3 (18.8)	4 (16.7)	5 (22.7)	8 (33.3)	26 (41.9)	0.012
In-hospital mortality	284 (42.1)	5 (31.2)	5 (20.8)	9 (40.9)	17 (70.8)	38 (61.3)	0.004
**Intubated patients (n = 580)**	(n = 455)	(n = 15)	(n = 18)	(n = 19)	(n = 18)	(n = 55)	
28-day mortality	148 (32.5)	5 (33.3)	4 (22.2)	7 (36.8)	5 (27.8)	23 (41.8)	0.323
ICU mortality	146 (32.1)	3 (20.0)	4 (22.2)	5 (26.3)	6 (33.3)	26 (47.3)	0.095
In-hospital mortality	225 (49.5)	5 (33.3)	5 (27.8)	9 (47.4)	13 (72.2)	35 (63.6)	0.043

*Note*: Data are presented as numbers (%).

*ICU* intensive care unit.

In the subgroup analysis of intubated patients, the in-hospital mortality rate was higher for patients with severe bronchiectasis than for the other patients (62.0% vs. 48.2%, *P* = 0.021, Table 2), and the rate increased with an increase in the number of lobes involved in bronchiectasis (*P* = 0.043, Table 3).

After adjusting for age, sex, BMI, presence of COPD, APACHE II score, SOFA score, and CCI score, patients with severe bronchiectasis had higher odds of 28-day mortality (OR 1.122, 95% CI 1.024–1.230), ICU mortality (OR 1.119, 95% CI 1.023–1.223), and in-hospital mortality (OR 1.208, 95% CI 1.092–1.337) (Table 4). The severe bronchiectasis group among intubated patients also showed higher odds of in-hospital mortality (OR 2.113, 95% CI 1.261–3.584).

**Table 4:** Logistic regression analysis for all-cause mortality.

	28-day mortality		ICU mortality		In-hospital mortality	
	Adjusted OR (95% CI)	*P-*value	Adjusted OR (95% CI)	*P-*value	Adjusted OR (95% CI)	*P-*value
**All patients (n = 758)**						
Age, year	1.000 (0.998–1.002)	0.908	0.999 (0.997–1.001)	0.318	1.000 (0.998–1.003)	0.890
Sex, female	1.018 (0.955–1.084)	0.586	1.021 (0.961–1.086)	0.499	1.007 (0.939–1.080)	0.838
BMI, kg/m^2^	1.005 (0.997–1.011)	0.209	1.001 (0.994–1.008)	0.790	1.003 (0.995–1.010)	0.518
COPD	1.020 (0.925–1.124)	0.697	0.974 (0.886–1.071)	0.593	1.012 (0.909–1.127)	0.829
APACHE II score	1.010 (1.006–1.013)	< 0.001	1.008 (1.004–1.011)	< 0.001	1.010 (1.006–1.014)	< 0.001
SOFA score	1.030 (1.020–1.039)	< 0.001	1.031 (1.022–1.040)	< 0.001	1.027 (1.017–1.037)	< 0.001
CCI score	1.009 (0.997–1.021)	0.129	1.008 (0.997–1.020)	0.158	1.017 (1.003–1.030)	0.015
Severe bronchiectasis	1.122 (1.024–1.230)	0.014	1.119 (1.023–1.223)	0.014	1.208 (1.092–1.337)	< 0.001
**Intubated patients (n = 539)**						
Age, year	1.000 (0.998–1.003)	0.869	0.996 (0.982–1.010)	0.570	1.006 (0.992–1.019)	0.412
Sex, female	1.043 (0.965–1.128)	0.290	1.266 (0.834–1.919)	0.267	1.132 (0.768–1.672)	0.532
BMI, kg/m^2^	1.004 (0.995–1.012)	0.419	1.005 (0.960–1.051)	0.845	1.008 (0.966–1.052)	0.718
COPD	1.020 (0.914–1.139)	0.724	0.944 (0.523–1.746)	0.851	1.109 (0.639–1.939)	0.713
APACHE II score	1.007 (1.003–1.012)	0.003	1.023 (0.998–1.049)	0.072	1.020 (0.997–1.045)	0.095
SOFA score	1.041 (1.029–1.052)	< 0.001	1.206 (1.137–1.282)	< 0.001	1.188 (1.124–1.259)	< 0.001
CCI score	1.009 (0.994–1.023)	0.255	1.047 (0.969–1.131)	0.240	1.071 (0.995–1.153)	0.067
Severe bronchiectasis	1.098 (0.990–1.219)	0.078	1.726 (0.999–2.960)	0.048	2.113 (1.261–3.584)	0.005

*APACHE II* Acute Physiology and Chronic Health Evaluation II, *BMI* body mass index, *CCI* Charlson comorbidity index, *CI* confidence interval, *OR* odds ratio.

### Survival analysis

**Figure 1:**
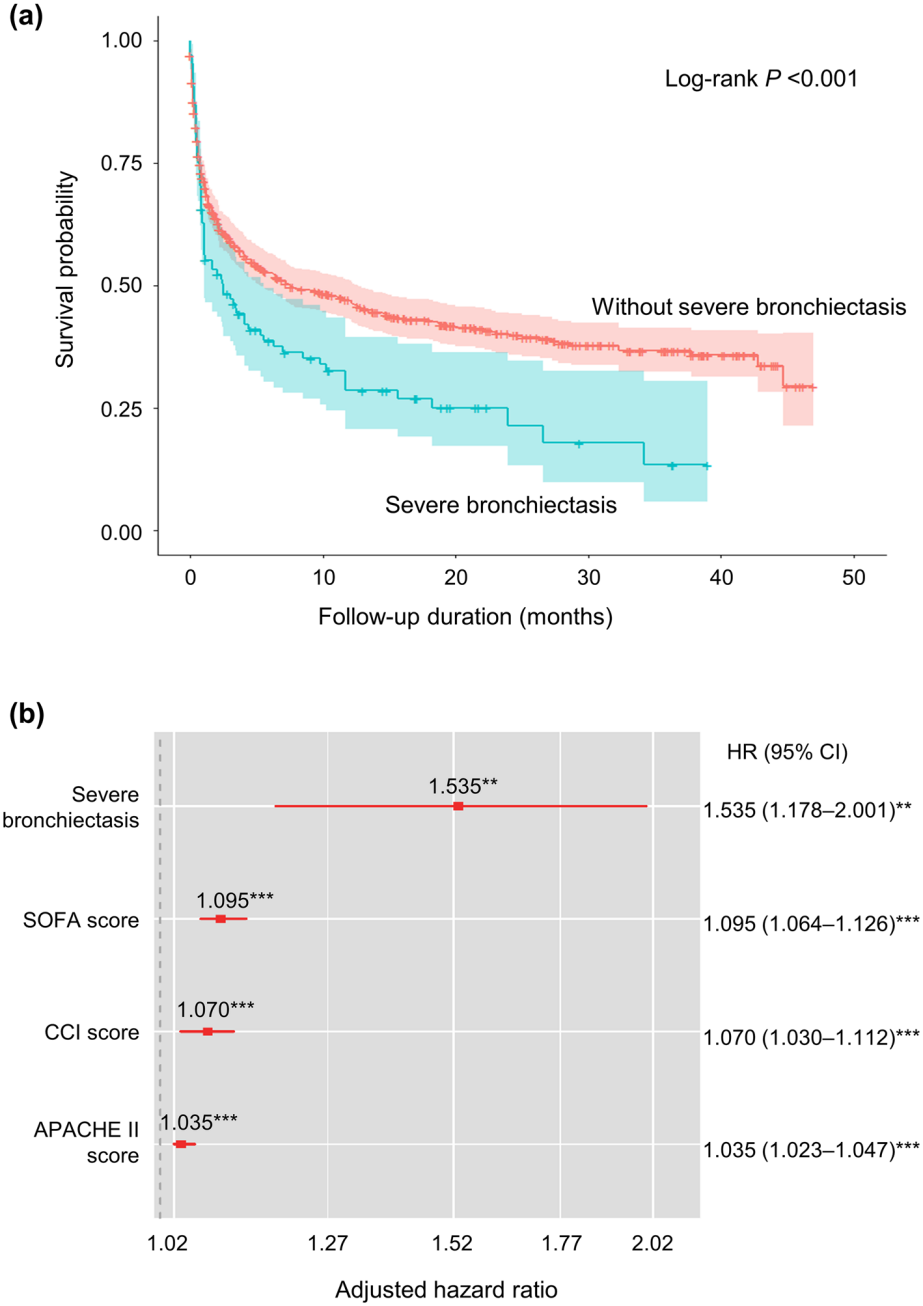
Survival analysis of the study group. (**a**) Kaplan–Meier curve and Log-rank test. (**b**) HRs of significant variables. Age, sex, BMI, and COPD were adjusted. Asterisks represent *P-*values: * < 0.05, ** < 0.01, *** < 0.001. *APACHE-II* Acute Physiology and Chronic Health Evaluation II, *BMI* body mass index, *CCI* Charlson comorbidity index, *CI* confidence interval, *COPD* chronic obstructive pulmonary disease, *HR* hazard ratio, *SOFA* sequential organ failure assessment.

**Figure 2:**
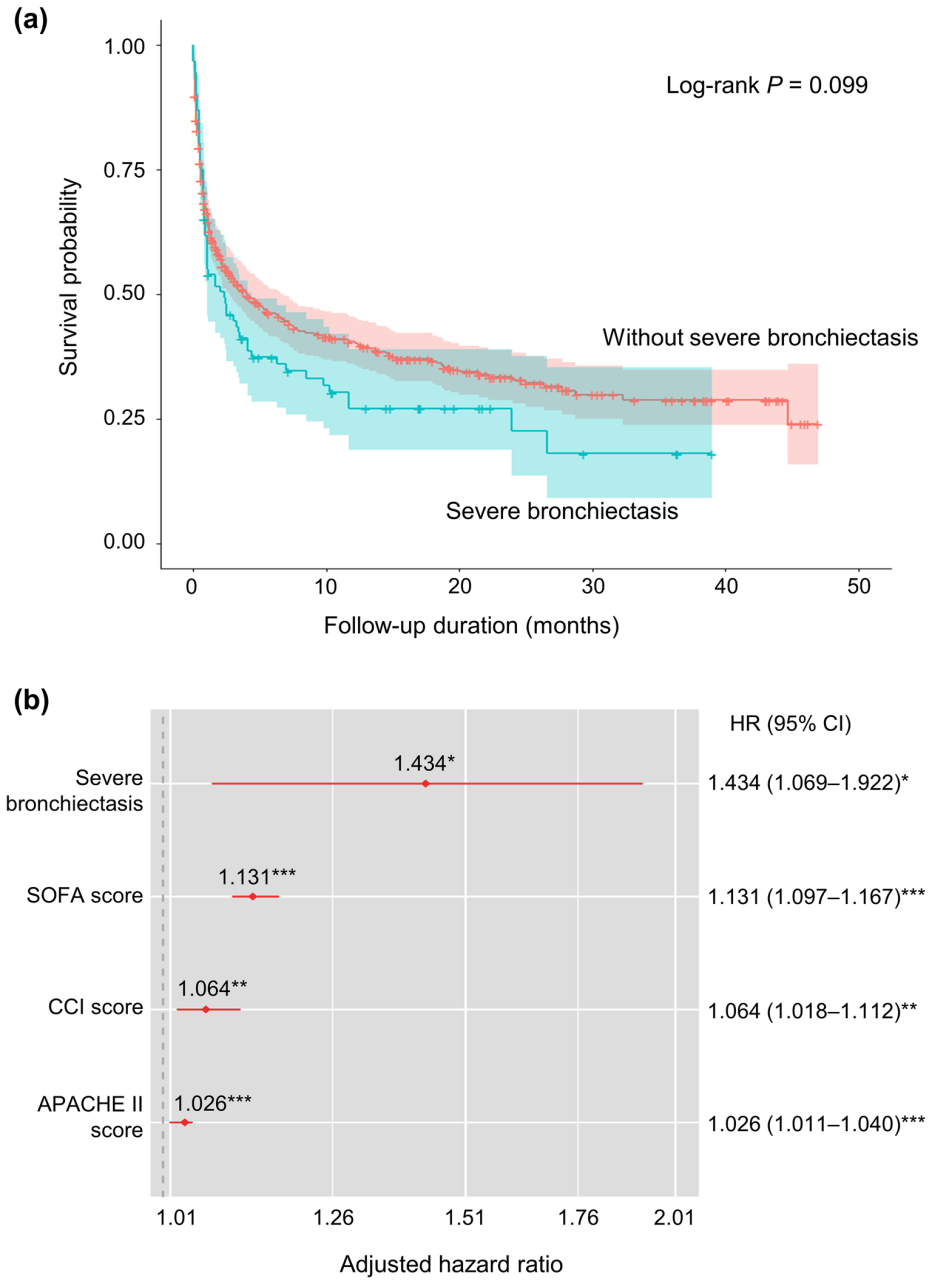
Survival analysis among the intubated patients. (**a**) Kaplan–Meier curve and Log-rank test. (**b**) HRs of significant variables. Age, sex, BMI, and COPD were adjusted. Asterisks represent *P-*values: * < 0.05, ** < 0.01, *** < 0.001. *APACHE-II* Acute Physiology and Chronic Health Evaluation II, *BMI* body mass index, *CCI* Charlson comorbidity index, *CI* confidence interval, *COPD* chronic obstructive pulmonary disease, *HR* hazard ratio, *SOFA* sequential organ failure assessment.

The severe bronchiectasis group showed a lower overall survival rate than did the other group (Log-rank *P* < 0.001, Fig. 1), and the adjusted HR from the Cox proportional-hazards model was 1.535 (95% CI 1.178–2.001). Among the intubated patients, the overall survival of the severe bronchiectasis group was similar to that of the control group (Log-rank *P* = 0.099, Fig. 2), but the adjusted HR was 1.434 (95% CI 1.069–1.922) in the Cox proportional-hazards model.

## Discussion

In our study, the prevalence of bronchiectasis and its impact on critically ill patients were presented. The crude prevalence rates were 18.0% for bronchiectasis and 13.1% for severe bronchiectasis among those admitted to the medical ICU. Patients with severe bronchiectasis required prolonged ICU and hospital stays; moreover, severe bronchiectasis was a risk factor of all-cause death during ICU and in-hospital admission, thus lowering the survival rate.

Bronchiectasis is a common respiratory condition. The prevalence of bronchiectasis differs geographically: (per 100,000 individuals) 67–566 cases in Europe^4, 18, 19^, 139 cases (age > 18 years) and 1,106 cases (age ≥ 65 years) in the US^3, 20^, and > 1,200 cases (age > 40 years) in China^21^. The variations in the prevalence estimates might be due to host factor susceptibilities, etiologic differences, and analyzed data sets^22^. However, in recent decades, the prevalence and incidence of bronchiectasis have increased in an age-dependent manner^3, 4, 18–21^.

Limited data were available on bronchiectasis among patients admitted in the ICU. Navaratnam et al.^10^ reported that 536 patients (0.1%) among 614,352 patients were admitted to 219 critical care units because of bronchiectasis from 2009 to 2013 in the UK. The number of ICU admissions owing to bronchiectasis increased annually by 8% (95% CI 2–15), while those due to COPD increased by 1% (95% CI 0.3–2). In our study, there were 18.0% and 13.1% of the patients with bronchiectasis and severe bronchiectasis, respectively, among the ICU-admitted patients. The high prevalence of bronchiectasis and severe bronchiectasis in our study might result from the high rate of respiratory failure and intubation. Since our study was conducted in the non-coronary medical ICU, frequent chest CT scans to evaluate thoracic lesions might have led to a high prevalence of bronchiectasis and severe bronchiectasis. In addition, Yang et al. reported from a national database study that lower family income and comorbid pulmonary conditions such as previous pulmonary tuberculosis were independently associated with bronchiectasis in Korea^23^. As Korea was one of the fastest developing countries in the late 1900s and still has an intermediate burden of tuberculosis^24^, these factors might have contributed to the high prevalence of bronchiectasis in this study.

Bronchiectasis patients usually have multiple comorbidities. A European international cohort study revealed that bronchiectasis patients had a median of four diseases (interquartile range 2–6, range 0–20), which significantly contributed to disease burden and mortality^6^. In our study, bronchiectasis also seemed to contribute to chronic medical conditions. The ICU mortality and in-hospital mortality were higher in patients with severe bronchiectasis, whereas 28-day mortality, a possible surrogate marker of acute stage severity, showed no difference between the two groups. Also, 28-day mortality and ICU mortality among the intubated patients were similar between those with and without severe bronchiectasis. In contrast, the duration of ICU and in-hospital stays were longer in patients with severe bronchiectasis regardless of intubation. Although initial comorbidities represented by CCI scores were similar between the two groups, severe bronchiectasis might have contributed to the underlying medical condition and provoked complications during the admission.

Prolonged ICU stay accelerates ICU-acquired weakness and increases health-related costs^25^. Moreover, bronchiectasis results in economic and psychological burdens, not only among patients, but also among caregivers^26–28^. ICU stays impose higher medical expenditures than those in general wards^29, 30^. In our study, we could not directly measure medical expenses; however, the duration of ICU and hospital stay were longer in the severe bronchiectasis group.

The strength of our study is that we simplified the severity of bronchiectasis as the number of affected lobes on chest CT scans and measured the impact of severe bronchiectasis on overall mortality. Several studies used the Bronchiectasis Severity Index^16^ or FACED score^17^ to grade disease severity. Both scores require spirometry results, dyspnea scale of the Medical Research Council, sputum culture for bacterial colonization, and chest CT scans. However, only the radiological extent of bronchiectasis was used in our study for evaluating the severity since critically ill patients often lack prior pulmonary function tests in actual practice. Additionally, incidental diagnosis of bronchiectasis on chest CT scans was common; therefore, we decided to use the radiological extent to reflect the severity in the ICU setting. We found that the radiological severity of three or more bronchiectatic lobes indicated a clinically useful predictor of all-cause mortality among critically ill patients.

Our study is subject to limitations. First, this was a single-center retrospective study. A prospective multi-center study is needed for confirming the impact of bronchiectasis on critically ill patients. Second, there may be selection bias as chest CT scans were acquired for 68.2% of all ICU-admitted patients. Third, the relationship between the number of bronchiectatic lobes and mortality could not be established. Since patients with bronchiectasis accounted for 18.0% of the study population, dividing them by the number of lobes resulted in insufficient numbers in each subgroup. Thus, further studies with a larger population would be required. Fourth, the impact of bronchiectasis on the specific cause of death could not be evaluated. Sin et al.^31^ reported that the increased risk of all-cause mortality in bronchiectasis patients was related to respiratory disease and lung cancer when compared with the control population in Korea. However, as ICU-admitted patients usually have multiple organ damage, it is hard to define the specific reason for mortality. Additionally, bronchiectasis might be an indirect cause of death. In our study, intubated patients with severe bronchiectasis showed an extended duration of ICU and hospital stays and higher in-hospital mortality despite the lower initial SOFA score. Therefore, severe bronchiectasis might be associated with a risk of all-cause death other than respiratory-related mortality. Fifth, we could not compare patients with bronchiectasis to patients with other respiratory diseases, because of the small number of patients with bronchiectasis exacerbation enrolled in this study. Sixth, the review of respiratory symptoms or prior colonization patterns of high-risk organisms was limited due to the retrospective design and the study setting, a tertiary referral center. Lastly, we did not specify the ICU admission diagnosis and only performed subgroup analysis in patients with respiratory failure requiring mechanical ventilation. Thus, exacerbation of bronchiectasis or superimposed pneumonia in sections of the study population might have confounded the results.

In conclusion, the prevalence of non-cystic fibrosis bronchiectasis was common in an ICU of a tertiary hospital in Korea. Severe bronchiectasis could be a risk factor for all-cause mortality during ICU and hospital stays, lowering the survival rate. The radiological extent of bronchiectasis could be a clinical predictor of poor prognosis. Since there are no clinical guidelines to manage patients with bronchiectasis in the ICU, further study would be needed.

## Data Availability

The datasets used and/or analyzed during the current study are available from the corresponding author on reasonable request.
